# Modelling of longitudinal data to predict cardiovascular disease risk: a methodological review

**DOI:** 10.1186/s12874-021-01472-x

**Published:** 2021-12-18

**Authors:** David Stevens, Deirdre A. Lane, Stephanie L. Harrison, Gregory Y. H. Lip, Ruwanthi Kolamunnage-Dona

**Affiliations:** 1 Liverpool Centre for Cardiovascular Science, University of Liverpool, Liverpool, L7 8TX UK; 2 Cardiovascular and Metabolic Medicine, Institute of Life Course and Medical Sciences, University of Liverpool, Liverpool, UK; 3 Department of Clinical Medicine, Aalborg University, Aalborg, Denmark; 4 Department of Health Data Science, Institute of Population Health, University of Liverpool, Liverpool, UK

**Keywords:** Cardiovascular disease, Longitudinal, Repeated measures, Risk prediction, Methodological review

## Abstract

**Objective:**

The identification of methodology for modelling cardiovascular disease (CVD) risk using longitudinal data and risk factor trajectories.

**Methods:**

We screened MEDLINE-Ovid from inception until 3 June 2020. MeSH and text search terms covered three areas: data type, modelling type and disease area including search terms such as “longitudinal”, “trajector*” and “cardiovasc*” respectively. Studies were filtered to meet the following inclusion criteria: longitudinal individual patient data in adult patients with ≥3 time-points and a CVD or mortality outcome. Studies were screened and analyzed by one author. Any queries were discussed with the other authors. Comparisons were made between the methods identified looking at assumptions, flexibility and software availability.

**Results:**

From the initial 2601 studies returned by the searches 80 studies were included. Four statistical approaches were identified for modelling the longitudinal data: 3 (4%) studies compared time points with simple statistical tests, 40 (50%) used single-stage approaches, such as including single time points or summary measures in survival models, 29 (36%) used two-stage approaches including an estimated longitudinal parameter in survival models, and 8 (10%) used joint models which modelled the longitudinal and survival data together. The proportion of CVD risk prediction models created using longitudinal data using two-stage and joint models increased over time.

**Conclusions:**

Single stage models are still heavily utilized by many CVD risk prediction studies for modelling longitudinal data. Future studies should fully utilize available longitudinal data when analyzing CVD risk by employing two-stage and joint approaches which can often better utilize the available data.

**Supplementary Information:**

The online version contains supplementary material available at 10.1186/s12874-021-01472-x.

## Background

Cardiovascular disease (CVD) is a leading cause of morbidity and mortality worldwide, accounting for 47 and 39% of deaths in females and males, respectively, in European Society of Cardiology member states [1]. Risk prediction models inform the understanding and management of CVD and have become an important part of clinical decision making. Many risk prediction models for CVD use one data point per patient (usually at baseline), such as the widely used Framingham Risk Score which predicts risk for coronary heart disease, [2] or QRISK3 which predicts risk of CVD in a subset of the UK population, and is widely used in CVD risk stratification in the UK [3]. These models use many variables at baseline including systolic blood pressure (SBP), total cholesterol, high-density lipoprotein cholesterol, or smoking status. As such, many cardiovascular risk prediction models do not account for measurement error or changes in risk factors over time [4, 5] which could lead to biased estimation. For example, SBP generally increases as people age, while diastolic blood pressure initially rises but starts decreasing after the age of 60 [6]. Further, as people age, they accumulate more risk factors. These complex and dynamic changes over time must be accounted for when modelling CVD risk to achieve the most robust possible risk prediction.

In risk prediction, longitudinal data permits the study of change in risk factors over time, accounting for within person-variance and usually provides an increase in power while reducing the number of patients needed [7]. However, analysis of longitudinal data adds complexity, such as dependence between observations, informatively censored or incomplete data and non-linear trajectories of longitudinal risk factors over time. Addressing these issues can add significant complexity and computational burden to the analysis.

The association between longitudinal measurements of blood pressure and risk of CVD has been studied using summaries such as time-averaged, cumulative, [8] trajectory patterns [9] and variability [10, 11]. However, less effort has been invested in modelling the complete record of longitudinal measurements, e.g. as time-varying covariates. Using summary measures in risk prediction models could be ineffective due to possible heterogeneity of variance for the summary measure. A review of risk prediction models covering the period 2009–2016 found that 46/117 (39.3%) studies considered longitudinal data, and only 9/117 (7.7%) studies included longitudinal data as time-varying covariates [12]. A more recent review of available methods adopted for harnessing longitudinal data in clinical risk prediction showed a further increase in the development of risk prediction models over the period 2009–2018 and identified seven different methodological frameworks [13].

The aim of this review was to conduct a comprehensive methodological evaluation of the estimation of risk for developing CVD in the general population, specifically targeting studies with a longitudinal design with three or more time-points, to allow for the trajectory of the longitudinal variable(s) to be modelled in predicting CVD risk.

## Material and methods

### Selection criteria

This review focused on risk prediction for CVD. Studies were included if they had a longitudinal design with data analyzed over at least three time points, where the outcome was a clinical diagnosis of a cardiovascular disease(s) or mortality. Cross-sectional, animal, and paediatric studies were excluded.

### Search strategy

**Fig. 1:**
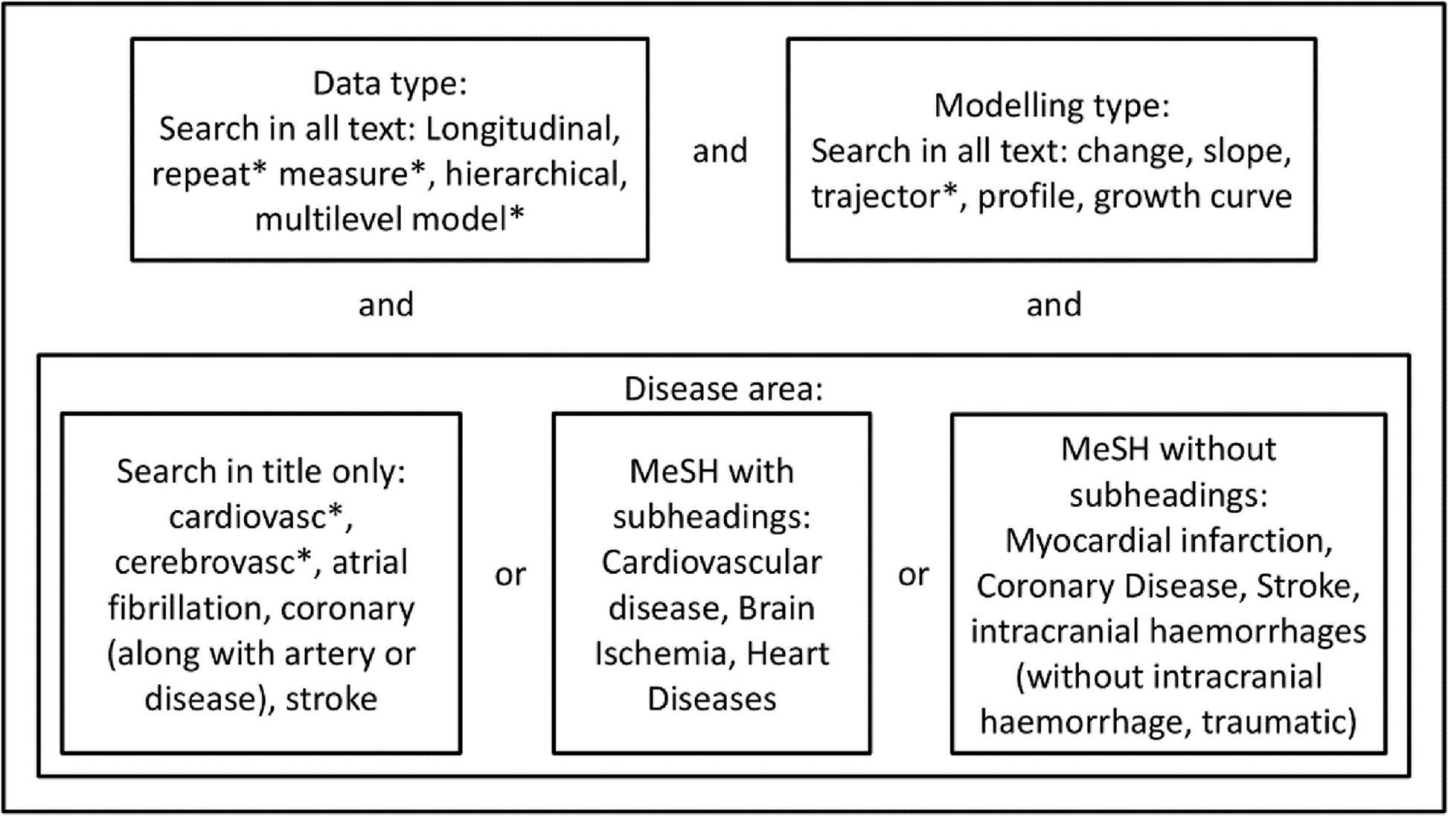
Summary of search strategy

MEDLINE-Ovid was searched from inception until 3 June 2020 with no language restrictions. Search terms used for data type and modelling type were “longitudinal, repeat* measure*, hierarchical, multilevel model*” and “change, slope, trajector*, profile, growth curve” respectively in all text. For disease area, the following search terms were used: “cardiovasc*, cerebrovasc*, atrial fibrillation, coronary (and artery or disease), stroke” in title, “cardiovascular disease, brain ischemia, heart diseases” in MeSH with subheadings or “myocardial infarction, coronary disease, stroke, intracranial hemorrhages (without intracranial hemorrhage, traumatic)” in MeSH without subheadings. The standardized search filter, along with the search approach and search terms are listed in Fig. 1 and Supplementary Table 1. Studies needed at least one term for data type, modelling type and disease area. Further, the reference lists of included studies were reviewed to identify any additional relevant articles.

Consideration of studies for inclusion followed a three-step process. First, titles were considered. Second, abstracts of potentially eligible studies were considered. Third, after abstract screening, the full-text articles were retrieved and assessed for eligibility. The first author (DS) completed the screening of studies and other authors were consulted to resolve any queries. Reasons for exclusion were recorded.

### Data extraction

The following information were extracted from each study: first author, year of publication, model type, dataset region, time period for data collection, age range, proportion of males, length of follow-up, number of patients, number of longitudinal time points, longitudinal and survival outcome data types, covariates adjusted for in longitudinal and survival models, survival and longitudinal outcomes, and characteristics of the statistical and modelling approaches used including assumptions, handling of missing data, model selection, and software used. Data extraction was conducted by the first author (DS), with other authors consulted to resolve any queries.

## Results

**Fig. 2:**
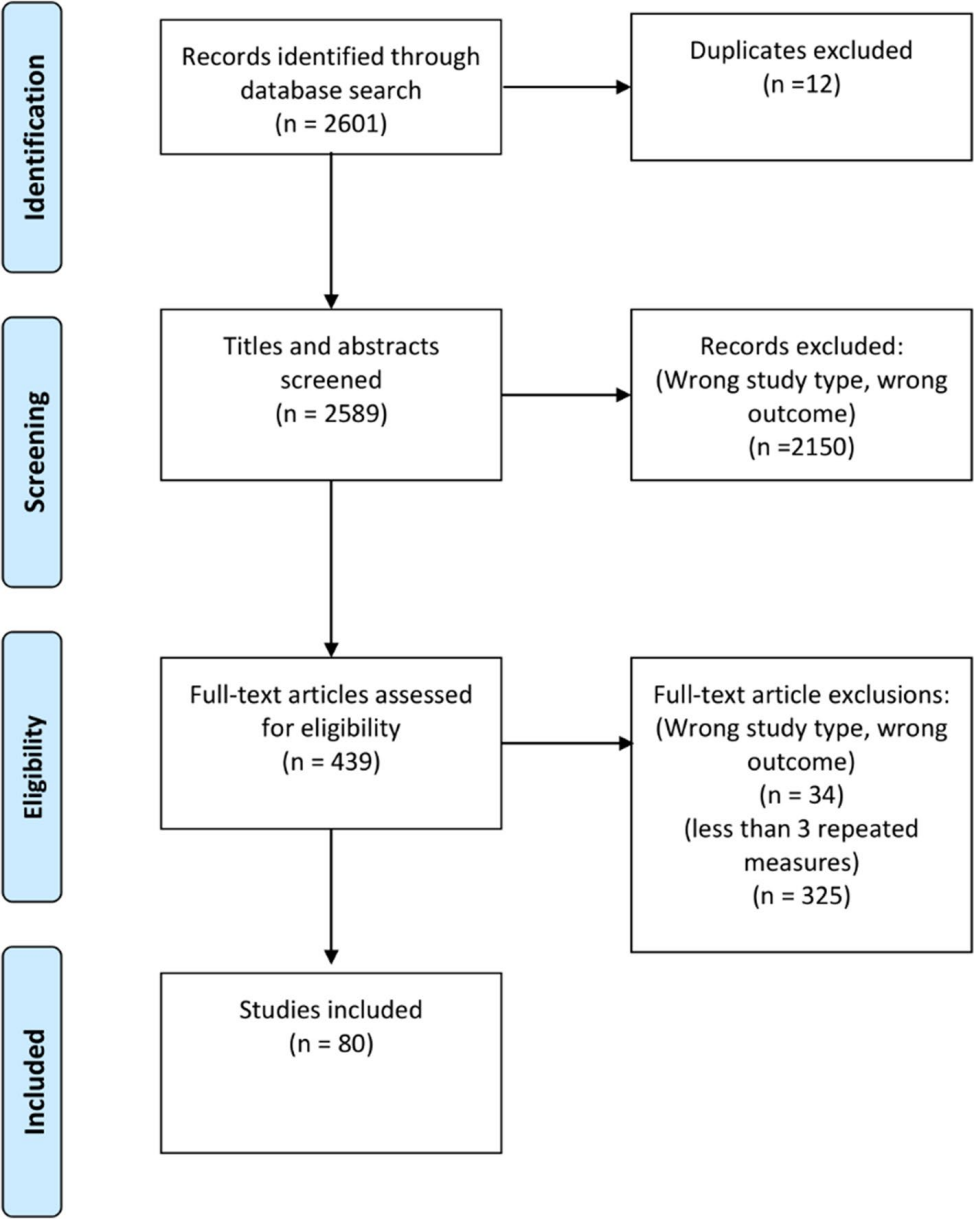
Flow chart of study selection

The searches returned 2601 studies with 12 duplicates (Fig. 2). Based on screening titles and abstracts, 2150 studies were excluded. The full texts were considered for 439 articles and a further 34 were excluded due to ≥1 of the following reasons: data not longitudinal, review article, data were summary measures rather than individual patient data, or non-CVD/mortality outcome. The number of repeated measures was assessed for 405 studies. A further 325 further studies were excluded due to having less than three repeated measures reported. Eighty studies were included in the review (Fig. 2) [14–93].

### General characteristics

Characteristics of the included studies are summarized in Table 1. Sixty (75%) studies reported analyses on large sample sizes (≥1000 patients). Exactly three longitudinal measurements were available in 27 (33.8%) studies, while 47 (58.8%) reported ≥3 data points with a mixture of median, mean or maximum number of longitudinal observations per patient; however, many studies did not utilize all available measurements. Follow-up lengths varied widely from 31 days [48] to 35 years, [50] with 29 (36.2%) reporting over a 10–20-year period. Patients were often followed up for survival after the last repeated measure, with 47 (58.8%) studies reporting a total follow-up of ≥10 years, while 31 (38.8%) reported a longitudinal outcome follow-up of ≥10 years. Over three-quarters (*n* = 65, 81.3%) were published after 2010, 15 studies (18.8%) were published prior to 2010. Data collection for many longitudinal datasets (*n* = 20, 25.0%) began in the 1980s, only 13 (16.2%) studies were from the 1990s, and about one-third were completed in the 2000s (*n* = 26, 32.5%).

**Table 1:** General characteristics of studies and outcomes included in the review

Model or study characteristic		Number of articles (%)	References
Number of patients	< 100	5 (6.3)	[15, 16, 21, 38, 58]
	100–999	13 (16.5)	[14, 26, 43, 45, 57, 61, 62, 64, 82, 85, 89, 90, 92]
	1000–9999	39 (49.4)	[17, 19, 23, 27, 28, 30–35, 37, 39–42, 44, 46, 47, 50, 53–56, 63, 66, 68, 70, 71, 73, 74, 76, 78, 79, 83, 86, 88, 91, 93]
	10,000+	21 (26.6)	[18, 20, 22, 24, 25, 29, 36, 49, 51, 52, 59, 60, 65, 67, 69, 72, 75, 77, 81, 84, 87]
	Not reported	1 (1.3)	[80]
Number of time points	Median 2	1 (1.2)	[90]
	3	27 (33.8)	[14–16, 20, 22, 24, 27–29, 31–33, 35, 36, 41, 53, 60, 61, 64, 65, 67, 69, 75, 78, 81, 86, 93]
	≥3 (median, mean or maximum)	47 (58.8)	[17–19, 21, 23, 30, 34, 37–39, 42–46, 48–52, 54–58, 62, 63, 66, 68, 70–74, 76, 77, 79, 80, 82–85, 87–89, 91, 92]
	Not reported	5 (6.2)	[25, 26, 40, 47, 59]
Follow-up for longitudinal and survival length (years)	< 5	16 (20.0)	[14–16, 21, 28, 32, 40, 43, 47, 48, 51, 57–59, 81, 87]
	5 to 10	17 (21.2)	[22, 24, 26, 27, 31, 38, 54, 55, 60, 61, 64, 65, 67, 72, 82, 84, 93]
	10 to 20	29 (36.2)	[18, 25, 29, 30, 33–35, 37, 41, 44, 45, 49, 62, 63, 69, 71, 74, 76–79, 83, 85, 86, 88–92]
	> 20	18 (22.5)	[17, 19, 20, 23, 36, 39, 42, 46, 50, 52, 53, 56, 66, 68, 70, 73, 75, 80]
Follow-up for longitudinal length (years)	< 5	24 (30.0)	[14–16, 21, 28, 32, 38, 40, 43, 47, 48, 51, 57–61, 64, 65, 67, 69, 71, 81, 87]
	5 to 10	25 (31.2)	[22, 24, 26, 27, 31, 33–35, 49, 53–56, 62, 72, 74, 76, 78, 79, 82–86, 93]
	10 to 20	23 (28.8)	[17–20, 25, 29, 30, 37, 41, 44, 45, 50, 63, 66, 70, 75, 77, 80, 88–92]
	> 20	8 (10.0)	[23, 36, 39, 42, 46, 52, 68, 73]
Follow-up for survival length (years)	< 5	19 (23.8)	[21, 24, 28, 32, 40, 43, 47, 48, 51, 57–61, 64, 67, 77, 81, 87]
	5 to 10	26 (32.5)	[19, 26, 27, 29, 31, 34, 35, 38, 41, 49, 54, 55, 65, 68–74, 76, 82–85, 93]
	10 to 20	23 (28.8)	[17, 18, 20, 25, 30, 36, 37, 39, 45, 56, 62, 63, 66, 75, 78–80, 86, 88–92]
	> 20	4 (5.0)	[23, 42, 50, 53]
	No survival analysis	8 (10.0)	[14–16, 22, 33, 44, 46, 52]
Time-period for start of data collection	1950s	2 (2.5)	[39, 80]
	1960s	6 (7.5)	[36, 50, 53, 56, 73, 78]
	1970s	5 (6.2)	[23, 42, 46, 66, 68]
	1980s	20 (25.0)	[17, 20, 26, 35, 37, 41, 44, 49, 52, 55, 62, 71, 74, 75, 79, 82, 85, 86, 88, 91]
	1990s	13 (16.2)	[18, 19, 27, 29, 30, 43, 45, 63, 70, 83, 89, 90, 92]
	2000s	26 (32.5)	[15, 22, 24, 25, 28, 31–34, 38, 47, 48, 51, 59–61, 65, 67, 69, 72, 76, 77, 81, 84, 87, 93]
	2010s	4 (5.0)	[14, 21, 58, 64]
	Not reported	4 (5.0)	[16, 40, 54, 57]
Decade of publication	Prior to 2000	8 (10.0)	[26, 43, 53–56, 78, 80]
	2000s	7 (8.8)	[15, 35, 49–52, 82]
	2010s	63 (78.8)	[14, 16, 18–25, 27–34, 36–42, 44–48, 57–68, 70–77, 79, 81, 83–93]
	2020	2 (2.5)	[17, 69]
Baseline Age - mean/median	< 40	5 (6.2)	[19, 26, 76, 77, 84]
	40–49	12 (15.0)	[16, 33, 39, 41, 44, 46, 47, 54, 68, 72, 86, 91]
	50–59	18 (22.5)	[14, 20, 23, 25, 28, 30, 34, 43, 45, 50, 56, 59, 60, 64–67, 69]
	60–69	17 (21.2)	[18, 21, 31, 32, 36–38, 40, 51, 57, 61, 63, 73, 79, 81, 92, 93]
	70–79	7 (8.8)	[17, 48, 71, 74, 85, 89, 90]
	> 80	2 (2.5)	[83, 87]
	Not reported	19 (23.8)	[15, 22, 24, 27, 29, 35, 42, 49, 52, 53, 55, 58, 62, 70, 75, 78, 80, 82, 88]
Region of dataset	Asia	16 (20.0)	[21, 22, 25, 34, 38, 58, 60, 61, 65, 67, 69, 76, 77, 80, 84, 88]
	Europe	22 (27.5)	[16, 20, 23, 24, 31, 36, 37, 39–41, 43, 44, 47, 49, 50, 52, 57, 75, 86, 87, 90, 91]
	International	3 (3.8)	[28, 32, 93]
	Middle East	3 (3.8)	[14, 45, 53]
	North America	33 (41.2)	[15, 17–19, 26, 29, 30, 33, 35, 42, 46, 48, 51, 54–56, 59, 62–64, 66, 68, 70, 71, 73, 74, 78, 79, 81–83, 85, 92]
	Australia & New Zealand	3 (3.8)	[27, 72, 89]
Males (%)	< 40	7 (8.8)	[16, 24, 46, 57, 74, 83, 91]
	40s	28 (35.0)	[14, 17–19, 25, 29, 31, 33, 35, 36, 40–42, 49, 52, 62, 64, 66, 68, 70, 71, 73, 75, 79, 82, 87–89]
	50s	7 (8.8)	[27, 43, 48, 77, 81, 84, 86]
	60–99	20 (25.0)	[20, 21, 28, 32, 34, 37, 38, 44, 45, 47, 58–61, 63, 65, 67, 69, 76, 93]
	All male	11 (13.8)	[23, 26, 39, 50, 53, 54, 56, 78, 85, 90, 92]
	Not reported	7 (8.8)	[15, 22, 30, 51, 55, 72, 80]
Survival outcome type	Binary	5 (6.2)	[17, 27, 30, 31, 64]
	Continuous	8 (10.0)	[14–16, 22, 33, 44, 46, 52]
	Rate	4 (5.0)	[23, 26, 37, 79]
	Time to event	63 (78.8)	[18–21, 24, 25, 28, 29, 32, 34–36, 38–43, 45, 47–51, 53–63, 65–78, 80–93]
Longitudinal outcome type	Binary	3 (3.8)	[24, 30, 55]
	Categorical	5 (6.2)	[20, 22, 65, 72, 73]
	Continuous	69 (86.2)	[14–19, 21, 23, 25–29, 31–33, 35, 36, 38–40, 42–54, 56–64, 66–71, 74–93]
	Ordinal	3 (3.8)	[34, 37, 41]
Survival analysis adjusted for age	Unadjusted	8 (10.0)	[21, 23, 30, 35, 57, 68, 86, 87]
	Yes, Stratified	3 (3.8)	[22, 54, 80]
	Yes, Baseline hazard	1 (1.2)	[39]
	Yes, Covariate	61 (76.2)	[17–20, 24–29, 31, 32, 34, 36–38, 40–43, 45, 47–51, 53, 55, 56, 58–67, 69–79, 81–85, 88–93]
	No survival analysis	7 (8.8)	[14–16, 33, 44, 46, 52]
Survival analysis adjusted for sex	Unadjusted	4 (5.0)	[21, 57, 58, 87]
	Single sex	12 (15.0)	[23, 26, 39, 50, 53, 54, 56, 78, 85, 90–92]
	Yes, separate models	9 (11.2)	[29, 30, 35, 42, 49, 75, 76, 82, 88]
	Yes, stratified	3 (3.8)	[22, 68, 80]
	Yes, covariate	45 (56.2)	[17–20, 24, 25, 27, 28, 31, 32, 34, 36–38, 40, 41, 43, 45, 47, 48, 51, 55, 59–67, 69–74, 77, 79, 81, 83, 84, 86, 89, 93]
	No survival analysis	7 (8.8)	[14–16, 33, 44, 46, 52]
Longitudinal Analysis adjusted for age	Unadjusted	30 (37.5)	[33, 44, 49, 57, 58, 60–67, 69, 71–75, 78–83, 85, 87, 89, 90, 93]
	Yes, covariate	17 (21.2)	[17, 18, 31, 46, 47, 52, 54, 59, 68, 70, 76, 77, 84, 86, 88, 91, 92]
	No longitudinal analysis	33 (41.2)	[14–16, 19–30, 32, 34–43, 45, 48, 50, 51, 53, 55, 56]
Longitudinal Analysis adjusted for sex	Unadjusted	28 (35.0)	[33, 44, 49, 57, 58, 60–69, 71–75, 79–83, 87, 89, 93]
	Single sex	6 (7.5)	[54, 78, 85, 90–92]
	Yes, separate models	4 (5.0)	[52, 70, 76, 88]
	Yes, covariate	9 (11.2)	[17, 18, 31, 46, 47, 59, 77, 84, 86]
	No longitudinal analysis	33 (41.2)	[14–16, 19–30, 32, 34–43, 45, 48, 50, 51, 53, 55, 56]
Disease area	Chronic kidney disease	1 (1.2)	[87]
	Cushing’s disease	1 (1.2)	[16]
	Cardiovascular disease	61 (76.2)	[14, 15, 17, 19–23, 26–42, 44, 45, 47–52, 54, 56–58, 60, 61, 64, 66–76, 78, 79, 81, 84–86, 88, 91–93]
	Diabetes	1 (1.2)	[25]
	Gout	1 (1.2)	[59]
	Hypertension	1 (1.2)	[63]
	Impaired sleep	1 (1.2)	[24]
	Mortality	5 (6.2)	[43, 62, 82, 83, 89]
	Systemic lupus erythematosus	1 (1.2)	[46]
	Stroke	7 (8.8)	[18, 53, 55, 65, 77, 80, 90]
Primary Outcome	Acute coronary syndrome	4 (5.0)	[30, 36, 60, 81]
	Atrial fibrillation	2 (2.5)	[25, 66]
	Cardiovascular mortality	7 (8.8)	[32, 39, 49–51, 54, 73]
	Cardiovascular Mortality/acute coronary syndrome/stroke	1 (1.2)	[93]
	Cardiovascular disease	36 (45.0)	[17, 19, 20, 23, 26, 27, 29, 31, 34, 35, 37, 38, 41, 42, 47, 56, 58, 59, 64, 68–72, 74–76, 78, 79, 84–86, 88, 89, 91, 92]
	Cardiovascular disease risk	8 (10.0)	[14–16, 22, 33, 44, 46, 52]
	Cardiovascular disease/cancer/mortality	1 (1.2)	[40]
	Cardiovascular disease/mortality	2 (2.5)	[21, 57]
	Hospitalization/heart failure/cardiovascular mortality	1 (1.2)	[28]
	Hypertension	1 (1.2)	[24]
	Mortality	9 (11.2)	[43, 45, 48, 55, 61–63, 82, 83]
	Stroke	8 (10.0)	[18, 53, 65, 67, 77, 80, 87, 90]
Population focus	Acute coronary syndrome	4 (5.0)	[21, 32, 45, 58]
	Atrial fibrillation and chronic kidney disease	1 (1.2)	[87]
	Chronic kidney disease	2 (2.5)	[43, 51]
	Cushing’s disease	1 (1.2)	[16]
	Cardiovascular disease	37 (46.2)	[15, 17, 19, 20, 23, 26, 27, 29, 33–35, 38, 40, 42, 44, 48, 53, 56, 57, 60–62, 65–71, 74, 78, 79, 81–83, 85, 88]
	Diabetes	3 (3.8)	[25, 64, 93]
	General population	27 (33.8)	[14, 18, 22, 24, 30, 31, 36, 37, 39, 41, 49, 50, 52, 54, 55, 63, 72, 73, 75–77, 80, 84, 86, 90–92]
	Gout	1 (1.2)	[59]
	Heart failure	1 (1.2)	[28]
	HIV	1 (1.2)	[47]
	Systemic lupus erythematosus	1 (1.2)	[46]
	Mental health	1 (1.2)	[89]

### Outcome data

Most (*n* = 63, 78.8%) studies reported disease outcomes as time-to-event or survival outcomes. Fewer studies examined disease outcomes as binary (*n* = 5, 6.2%), continuous (*n* = 8, 10.0%) or rates (*n* = 4, 5.0%). Most (*n* = 69, 86.2%) longitudinal outcomes were continuous; other longitudinal outcome types were binary (*n* = 3, 3.8%), categorical (n = 5, 6.2%), or ordinal (n = 3, 3.8%).

### Adjusting for covariates

Sixty-one studies (76.2%) adjusted for age and 45 (56.2%) adjusted for sex as covariates in their survival analysis, while four (5.0%) stratified by age and three (3.8%) for sex. Nine (11.2%) studies analyzed data separately for each sex. Seventeen (21.2%) longitudinal analyses were adjusted for age, while 30 (37.5%) were not. Sex was adjusted for as a covariate in 9 (11.2%) longitudinal analyses. Four (5.0%) studies analyzed longitudinal data separately by sex, and 28 (35.0%) did not adjust for sex.

### Statistical analysis

This review has identified a variety of statistical analysis methods that have been incorporated to analyze time-to-event and longitudinal outcome data. Three (3.8%) used a simple statistical test [14–16]. For example, Albani et al. [16] used the Wilcoxon signed rank test to compare two risk scores (the Framingham Risk Score and an atherosclerotic cardiovascular disease risk score) before treatment with pasireotide and 6 and 12 months after treatment. Other statistical approaches for modelling CVD risk using longitudinal data can be divided into three categories: 1) single-stage approaches including basic summary measures, 40 (50.0%), [17–56] 2) two-stage approaches using an estimated longitudinal parameter as a covariate in a survival outcome model, 29 (36.3%), [57–85] and 3) joint models fitting longitudinal and survival data simultaneously, 8 (10.0%) [86–93].

### Characteristics of included studies

**Fig. 3:**
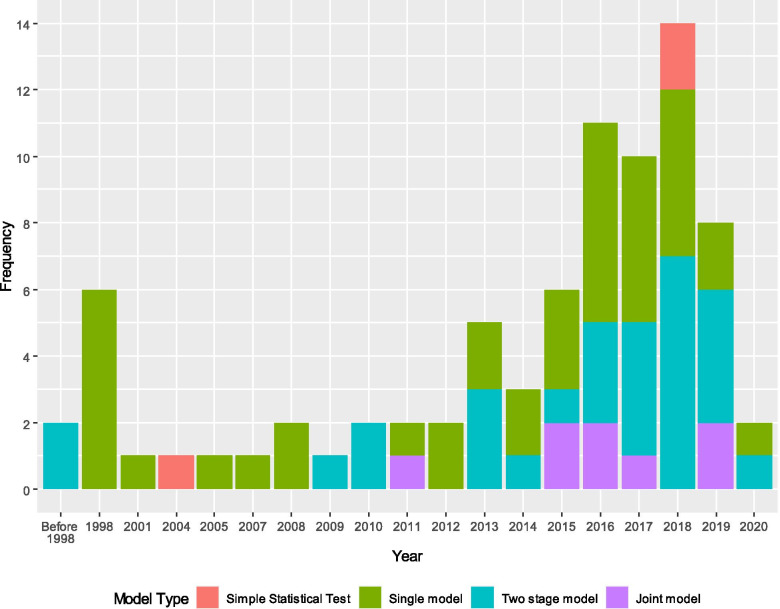
Stacked bar chart showing the frequency of the statistical model types by year

The characteristics of the included studies by different modelling approaches is shown in Table 2. Joint models have been fitted on smaller datasets with only one study using a joint model on a dataset of over 10,000 patients [87]. A larger proportion of two-stage or joint models had patients with a variable number of time points included compared to single-stage approaches (24/37 (64.9%) vs. 23/40 (57.5%), respectively). Five (6.3%) studies did not report the number of time points used in their analyses. Two-stage approaches were used on 10/16 (62.5%) datasets collected in Asia but only in 2/22 (9.1%) on European datasets. The longitudinal analysis in two-stage approaches rarely adjusted for age or sex, with adjustments made in 6/29 (20.7%) and 7/29 (24.1%), respectively. The frequency of studies using each model type over time is shown in Fig. 3. Since 2010, a substantial increase in the number of papers using two-stage approaches was observed with 26/65 (40.0%) using them after 2010 vs. 3/15 (20.0%) before. Use of joint models also commenced later that decade with only one study before 2015.

**Table 2:** Summary of characteristics of studies included in the review by model type

Model or study characteristic		No of papers n (%)	Simple statistical tests n (%)	Single model^a^ n (%)	Two-stage model^b^ n (%)	Joint model^c^ n (%)	Complete case analysis n (%)
Number of patients	< 100	5 (6.3)	2 (66.7)	2 (5.0)	1 (3.4)	0 (0.0)	5 (100.0)
	100–999	13 (16.5)	1 (33.3)	3 (7.5)	6 (20.7)	3 (37.5)	11 (84.6)
	1000–9999	39 (49.4)	0 (0.0)	24 (60.0)	11 (37.9)	4 (50.0)	29 (74.4)
	10,000+	21 (26.6)	0 (0.0)	10 (25.0)	10 (34.5)	1 (12.5)	18 (85.7)
	Not reported	1 (1.3)	0 (0.0)	0 (0.0)	1 (3.4)	0 (0.0)	1 (100.0)
Number of time points	Median 2	1 (1.2)	0 (0.0)	0 (0.0)	0 (0.0)	1 (12.5)	0 (0.0)
	3	27 (33.8)	3 (100.0)	13 (32.5)	9 (31.0)	2 (25.0)	21 (77.8)
	≥3 (median, mean or maximum)	47 (58.8)	0 (0.0)	23 (57.5)	19 (65.5)	5 (62.5)	39 (83.0)
	Not reported	5 (6.2)	0 (0.0)	4 (10.0)	1 (3.4)	0 (0.0)	5 (100.0)
Follow-up for longitudinal and survival length (years)	< 5	16 (20.0)	3 (100.0)	8 (20.0)	4 (13.8)	1 (12.5)	14 (87.5)
	5 to 10	17 (21.2)	0 (0.0)	8 (20.0)	8 (27.6)	1 (12.5)	15 (88.2)
	10 to 20	29 (36.2)	0 (0.0)	12 (30.0)	11 (37.9)	6 (75.0)	23 (79.3)
	> 20	18 (22.5)	0 (0.0)	12 (30.0)	6 (20.7)	0 (0.0)	13 (72.2)
Follow-up for longitudinal length (years)	< 5	24 (30.0)	3 (100.0)	9 (22.5)	11 (37.9)	1 (12.5)	21 (87.5)
	5 to 10	25 (31.2)	0 (0.0)	13 (32.5)	10 (34.5)	2 (25.0)	23 (92.0)
	10 to 20	23 (28.8)	0 (0.0)	12 (30.0)	6 (20.7)	5 (62.5)	16 (69.6)
	> 20	8 (10.0)	0 (0.0)	6 (15.0)	2 (6.9)	0 (0.0)	5 (62.5)
Follow-up for survival length (years)	< 5	19 (23.8)	0 (0.0)	9 (22.5)	9 (31.0)	1 (12.5)	16 (84.2)
	5 to 10	26 (32.5)	0 (0.0)	12 (30.0)	13 (44.8)	1 (12.5)	23 (88.5)
	10 to 20	23 (28.8)	0 (0.0)	10 (25.0)	7 (24.1)	6 (75.0)	16 (69.6)
	> 20	4 (5.0)	0 (0.0)	4 (10.0)	0 (0.0)	0 (0.0)	2 (50.0)
	No survival analysis	8 (10.0)	3 (100.0)	5 (12.5)	0 (0.0)	0 (0.0)	8 (100.0)
Time-period for start of data collection	1950s	2 (2.5)	0 (0.0)	1 (2.5)	1 (3.4)	0 (0.0)	1 (50.0)
	1960s	6 (7.5)	0 (0.0)	4 (10.0)	2 (6.9)	0 (0.0)	6 (100.0)
	1970s	5 (6.2)	0 (0.0)	3 (7.5)	2 (6.9)	0 (0.0)	3 (60.0)
	1980s	20 (25.0)	0 (0.0)	10 (25.0)	7 (24.1)	3 (37.5)	16 (80.0)
	1990s	13 (16.2)	0 (0.0)	7 (17.5)	3 (10.3)	3 (37.5)	9 (69.2)
	2000s	26 (32.5)	1 (33.3)	12 (30.0)	11 (37.9)	2 (25.0)	22 (84.6)
	2010s	4 (5.0)	1 (33.3)	1 (2.5)	2 (6.9)	0 (0.0)	4 (100.0)
	Not reported	4 (5.0)	1 (33.3)	2 (5.0)	1 (3.4)	0 (0.0)	4 (100.0)
Decade of publication	Prior to 2000	8 (10.0)	0 (0.0)	6 (15.0)	2 (6.9)	0 (0.0)	8 (100.0)
	2000s	7 (8.8)	1 (33.3)	5 (12.5)	1 (3.4)	0 (0.0)	6 (85.7)
	2010s	63 (78.8)	2 (66.7)	28 (70.0)	25 (86.2)	8 (100.0)	49 (77.8)
	2020	2 (2.5)	0 (0.0)	1 (2.5)	1 (3.4)	0 (0.0)	2 (100.0)
Baseline Age - mean/median	< 40	5 (6.2)	0 (0.0)	2 (5.0)	3 (10.3)	0 (0.0)	5 (100.0)
	40–49	12 (15.0)	1 (33.3)	7 (17.5)	2 (6.9)	2 (25.0)	8 (66.7)
	50–59	18 (22.5)	1 (33.3)	10 (25.0)	7 (24.1)	0 (0.0)	13 (72.2)
	60–69	17 (21.2)	0 (0.0)	9 (22.5)	6 (20.7)	2 (25.0)	14 (82.4)
	70–79	7 (8.8)	0 (0.0)	2 (5.0)	3 (10.3)	2 (25.0)	6 (85.7)
	> 80	2 (2.5)	0 (0.0)	0 (0.0)	1 (3.4)	1 (12.5)	2 (100.0)
	Not reported	19 (23.8)	1 (33.3)	10 (25.0)	7 (24.1)	1 (12.5)	17 (89.5)
Data Region	Asia	16 (20.0)	0 (0.0)	5 (12.5)	10 (34.5)	1 (12.5)	15 (93.8)
	Europe	22 (27.5)	1 (33.3)	15 (37.5)	2 (6.9)	4 (50.0)	14 (63.6)
	International	3 (3.8)	0 (0.0)	2 (5.0)	0 (0.0)	1 (12.5)	2 (66.7)
	Middle East	3 (3.8)	1 (33.3)	2 (5.0)	0 (0.0)	0 (0.0)	2 (66.7)
	North America	33 (41.2)	1 (33.3)	15 (37.5)	16 (55.2)	1 (12.5)	29 (87.9)
	Australia & NZ	3 (3.8)	0 (0.0)	1 (2.5)	1 (3.4)	1 (12.5)	3 (100.0)
Males (%)	< 40	7 (8.8)	1 (33.3)	2 (5.0)	3 (10.3)	1 (12.5)	6 (85.7)
	40s	28 (35.0)	1 (33.3)	14 (35.0)	10 (34.5)	3 (37.5)	24 (85.7)
	50s	7 (8.8)	0 (0.0)	3 (7.5)	3 (10.3)	1 (12.5)	6 (85.7)
	60–99	20 (25.0)	0 (0.0)	10 (25.0)	9 (31.0)	1 (12.5)	16 (80.0)
	All male	11 (13.8)	0 (0.0)	7 (17.5)	2 (6.9)	2 (25.0)	8 (72.7)
	Not reported	7 (8.8)	1 (33.3)	4 (10.0)	2 (6.9)	0 (0.0)	5 (71.4)
Survival outcome type	Binary	5 (6.2)	0 (0.0)	4 (10.0)	1 (3.4)	0 (0.0)	3 (60.0)
	Continuous	8 (10.0)	3 (100.0)	5 (12.5)	0 (0.0)	0 (0.0)	8 (100.0)
	Rate	4 (5.0)	0 (0.0)	3 (7.5)	1 (3.4)	0 (0.0)	3 (75.0)
	Time to event	63 (78.8)	0 (0.0)	28 (70.0)	27 (93.1)	8 (100.0)	51 (81.0)
Longitudinal outcome type	Binary	3 (3.8)	0 (0.0)	3 (7.5)	0 (0.0)	0 (0.0)	2 (66.7)
	Categorical	5 (6.2)	0 (0.0)	2 (5.0)	3 (10.3)	0 (0.0)	4 (80.0)
	Continuous	69 (86.2)	3 (100.0)	32 (80.0)	26 (89.7)	8 (100.0)	57 (82.6)
	Ordinal	3 (3.8)	0 (0.0)	3 (7.5)	0 (0.0)	0 (0.0)	2 (66.7)
Survival analysis adjusted for age	Unadjusted	8 (10.0)	0 (0.0)	4 (10.0)	2 (6.9)	2 (25.0)	5 (62.5)
	Yes, Stratified	3 (3.8)	0 (0.0)	2 (5.0)	1 (3.4)	0 (0.0)	3 (100.0)
	Yes, Baseline hazard	1 (1.2)	0 (0.0)	1 (2.5)	0 (0.0)	0 (0.0)	0 (0.0)
	Yes, Covariate	61 (76.2)	0 (0.0)	29 (72.5)	26 (89.7)	6 (75.0)	50 (82.0)
	No survival analysis	7 (8.8)	3 (100.0)	4 (10.0)	0 (0.0)	0 (0.0)	7 (100.0)
Survival analysis adjusted for sex	Unadjusted	4 (5.0)	0 (0.0)	1 (2.5)	2 (6.9)	1 (12.5)	4 (100.0)
	Single sex	12 (15.0)	0 (0.0)	7 (17.5)	2 (6.9)	3 (37.5)	8 (66.7)
	Yes, separate models	9 (11.2)	0 (0.0)	5 (12.5)	3 (10.3)	1 (12.5)	7 (77.8)
	Yes, stratified	3 (3.8)	0 (0.0)	1 (2.5)	2 (6.9)	0 (0.0)	3 (100.0)
	Yes, covariate	45 (56.2)	0 (0.0)	22 (55.0)	20 (69.0)	3 (37.5)	36 (80.0)
	No survival analysis	7 (8.8)	3 (100.0)	4 (10.0)	0 (0.0)	0 (0.0)	7 (100.0)
Longitudinal Analysis adjusted for age	Unadjusted	30 (37.5)	0 (0.0)	3 (7.5)	23 (79.3)	4 (50.0)	28 (93.3)
	Yes, covariate	17 (21.2)	0 (0.0)	7 (17.5)	6 (20.7)	4 (50.0)	13 (76.5)
	No longitudinal analysis	33 (41.2)	3 (100.0)	30 (75.0)	0 (0.0)	0 (0.0)	24 (72.7)
Longitudinal Analysis adjusted for sex	Unadjusted	28 (35.0)	0 (0.0)	3 (7.5)	22 (75.9)	3 (37.5)	27 (96.4)
	Single sex	6 (7.5)	0 (0.0)	1 (2.5)	2 (6.9)	3 (37.5)	4 (66.7)
	Yes, separate models	4 (5.0)	0 (0.0)	1 (2.5)	2 (6.9)	1 (12.5)	3 (75.0)
	Yes, covariate	9 (11.2)	0 (0.0)	5 (12.5)	3 (10.3)	1 (12.5)	7 (77.8)
	No longitudinal analysis	33 (41.2)	3 (100.0)	30 (75.0)	0 (0.0)	0 (0.0)	24 (72.7)
Disease area	Chronic kidney disease	1 (1.2)	0 (0.0)	0 (0.0)	0 (0.0)	1 (12.5)	1 (100.0)
	Cushing’s disease	1 (1.2)	1 (33.3)	0 (0.0)	0 (0.0)	0 (0.0)	1 (100.0)
	Cardiovascular disease	61 (76.2)	2 (66.7)	33 (82.5)	21 (72.4)	5 (62.5)	47 (77.0)
	Diabetes	1 (1.2)	0 (0.0)	1 (2.5)	0 (0.0)	0 (0.0)	1 (100.0)
	Gout	1 (1.2)	0 (0.0)	0 (0.0)	1 (3.4)	0 (0.0)	1 (100.0)
	Hypertension	1 (1.2)	0 (0.0)	0 (0.0)	1 (3.4)	0 (0.0)	1 (100.0)
	Impaired sleep	1 (1.2)	0 (0.0)	1 (2.5)	0 (0.0)	0 (0.0)	1 (100.0)
	Mortality	5 (6.2)	0 (0.0)	1 (2.5)	3 (10.3)	1 (12.5)	5 (100.0)
	Systemic lupus erythematosus	1 (1.2)	0 (0.0)	1 (2.5)	0 (0.0)	0 (0.0)	1 (100.0)
	Stroke	7 (8.8)	0 (0.0)	3 (7.5)	3 (10.3)	1 (12.5)	6 (85.7)
Primary Outcome	Acute coronary syndrome	4 (5.0)	0 (0.0)	2 (5.0)	2 (6.9)	0 (0.0)	2 (50.0)
	Atrial fibrillation	2 (2.5)	0 (0.0)	1 (2.5)	1 (3.4)	0 (0.0)	2 (100.0)
	Cardiovascular mortality	7 (8.8)	0 (0.0)	6 (15.0)	1 (3.4)	0 (0.0)	4 (57.1)
	Cardiovascular mortality/acute coronary syndrome/stroke	1 (1.2)	0 (0.0)	0 (0.0)	0 (0.0)	1 (12.5)	1 (100.0)
	Cardiovascular disease	36 (45.0)	0 (0.0)	16 (40.0)	15 (51.7)	5 (62.5)	28 (77.8)
	Cardiovascular disease risk	8 (10.0)	3 (100.0)	5 (12.5)	0 (0.0)	0 (0.0)	8 (100.0)
	Cardiovascular disease/cancer/mortality	1 (1.2)	0 (0.0)	1 (2.5)	0 (0.0)	0 (0.0)	1 (100.0)
	Cardiovascular disease/mortality	2 (2.5)	0 (0.0)	1 (2.5)	1 (3.4)	0 (0.0)	2 (100.0)
	Hospitalization/heart failure/cardiovascular mortality	1 (1.2)	0 (0.0)	1 (2.5)	0 (0.0)	0 (0.0)	1 (100.0)
	Hypertension	1 (1.2)	0 (0.0)	1 (2.5)	0 (0.0)	0 (0.0)	1 (100.0)
	Mortality	9 (11.2)	0 (0.0)	4 (10.0)	5 (17.2)	0 (0.0)	8 (88.9)
	Stroke	8 (10.0)	0 (0.0)	2 (5.0)	4 (13.8)	2 (25.0)	7 (87.5)
Population focus	Acute coronary syndrome	4 (5.0)	0 (0.0)	3 (7.5)	1 (3.4)	0 (0.0)	2 (50.0)
	Atrial fibrillation and chronic kidney disease	1 (1.2)	0 (0.0)	0 (0.0)	0 (0.0)	1 (12.5)	1 (100.0)
	Chronic kidney disease	2 (2.5)	0 (0.0)	2 (5.0)	0 (0.0)	0 (0.0)	1 (50.0)
	Cushing’s disease	1 (1.2)	1 (33.3)	0 (0.0)	0 (0.0)	0 (0.0)	1 (100.0)
	Cardiovascular disease	37 (46.2)	1 (33.3)	17 (42.5)	18 (62.1)	1 (12.5)	32 (86.5)
	Diabetes	3 (3.8)	0 (0.0)	1 (2.5)	1 (3.4)	1 (12.5)	3 (100.0)
	General population	27 (33.8)	1 (33.3)	14 (35.0)	8 (27.6)	4 (50.0)	20 (74.1)
	Gout	1 (1.2)	0 (0.0)	0 (0.0)	1 (3.4)	0 (0.0)	1 (100.0)
	Heart failure	1 (1.2)	0 (0.0)	1 (2.5)	0 (0.0)	0 (0.0)	1 (100.0)
	HIV	1 (1.2)	0 (0.0)	1 (2.5)	0 (0.0)	0 (0.0)	1 (100.0)
	Systemic lupus erythematosus	1 (1.2)	0 (0.0)	1 (2.5)	0 (0.0)	0 (0.0)	1 (100.0)
	Mental health	1 (1.2)	0 (0.0)	0 (0.0)	0 (0.0)	1 (12.5)	1 (100.0)

^a^ One model is fitted e.g. fitted a Cox proportional hazards (PH) model for the survival outcome

^b^ Two models are fitted separately; one model was fitted to summarise longitudinal data or estimate the time effect, and then used that information in the model for disease or survival outcome. e.g. fitted a linear regression for longitudinal measurements for each individual, and then the estimated slopes were used in a Cox PH model

^c^ Longitudinal and survival outcomes are fitted simultaneously

A complete case analysis was used in 65/80 (81.3%) studies, more often in smaller (< 1000, 16/18, 88.9%) and very large (> 10,000, 18/21, 85.7%) cohorts than medium-sized studies (1000–9999, 29/39, 74.4%) and those with a variable number of time-points (39/48, 81.3%) compared with exactly three time points (21/27, 77.8%). In addition, those with shorter follow-ups (< 10 years, 19/33, 57.6%) were more likely to use a complete case analysis. The methods used for handling missing data included multiple imputation (*n* = 6), single imputations (*n* = 3), last observation carried forward (*n* = 2) and indicators for missing variables (n = 2).

### Single-stage approaches

A single-stage approach was used in 40 (50%) studies [17–56] (Table 3). The most common risk prediction model for single-stage models was the Cox proportional hazards (PH) model (*n* = 25, 62.5%) [94]. The model assumes a proportional effect on the hazard; the PH assumption should be checked, either by including time-varying coefficients or by a variety of graphical testing methods, such as Schoenfeld residual plots and log-log plots. Only 9/25 (62.5%) of articles utilizing Cox PH models as a single-stage approach stated that the PH assumption was checked [95].

**Table 3:** Summary of single-stage models used to incorporate longitudinal data in survival models

Method N(%)[refs]	Longitudinal outcome type	Disease outcome type	How the longitudinal data were used in the analysis N (%) [refs]	Reason for the use of method	Assumptions	Pros	Cons
**Single-stage approaches (*n*** **= 40)**							
Cox model, N = 25 (62.5) [18, 19, 21, 25, 28, 29, 32, 34–36, 38, 39, 41–43, 45, 47, 49–51, 53–57]	Continuous, Categorical	Time to event	Baseline only, *N* = 7 (17.5) [18, 21, 24, 43, 50, 53, 54] Continuous, N = 6 (15.0) [18, 21, 43, 50, 53, 54] Categorised, N = 2 (5.0) [24, 54]	To clinically relevant time point to be used for prediction	PH	Simple method	Dependence between measurement times is ignored
	Continuous	Time to event	Change from baseline, N = 3 (7.5) [28, 35, 38]	To incorporate change over time	PH; Change is linear	Incorporates more than one time point	Only looks at pairs of time points
	Continuous	Time to event	Slope calculated manually, N = 3 (7.5) [25, 29, 32]	To incorporate constant change in the survival model	PH; Change is linear	Incorporates more than one time point	Only looks at pairs of time points
	Continuous	Time to event	Average (categorized before use),^a^ *N* = 1 (2.5) [36]	To incorporate the average change over time	PH; Constant between time points; Change is linear	Incorporates the average impact over time	Interpretation unclear
	Continuous, Categorical	Time to event	Time-dependent covariate, *N* = 6 (15.0) [39, 42, 45, 47, 49, 51, 55]	To incorporate change in exposure variable over time	PH; Change is constant between two consecutive time points; Longitudinal data are measured without error	Incorporates time-varying measures over the follow-up period	Computationally slower as compared to time-fixed covariates; Computationally infeasible if the longitudinal outcome is measured at different time points for different individuals; Interpretation is difficult; Can lead to great overfitting of the data; must be used with caution
	Continuous	Time to event	Summary measures(Standard deviation, number of drops between observations), N = 1 (2.5) [19]	To incorporate variability summaries of the longitudinal data	PH	Incorporates variability of measures into the model	Summary measures fairly specific to dataset
	Continuous, Categorical	Time to event	Change in category between first and last time-point categorized, N = 2 (5.0) [34, 41] Change in continuous variable between time points categorized with manually defined cut-offs, *N* = 1 (2.5) [56]	To summarise trajectories in an interpretable way	PH	Results interpretable	Groups manually selected based on data which could lead to bias
Hierarchical Cox model to adjust for multiple studies, N = 1 (2.5) [20]	Continuous	Time to event	Continuous measurements categorized. Multiple time points also categorised as consistent/non-consistent, *N* = 1 (2.5) [20]	To summarise trajectories in an interpretable way adjusting for combining multiple studies	PH	Results interpretable; Adjusts for use of multiple studies	Groups manually selected based on data which could lead to bias
Logistic Regression, N = 3 (7.5) [30, 31, 48]	Continuous	Binary	Baseline only, N = 1 (2.5) [31]	Allows clinically relevant time point to be used for prediction	Not applicable	Simple method	Dependence between measurement times is ignored
	Categorical	Binary	Separate time points, *N* = 1 (2.5) [30]	To include all predictive values in model	Not applicable	Simple method	Caution needed for multicollinearity
	Continuous	Binary	Summaries of repeated measures • Standard deviation • Mean • Mean change from baseline • Average daily risk range^b^ • Range *N* = 1 (2.5) [48]	Includes different measures of variation	Not applicable	Simple method	Interpretation of different summary measures non-trivial
GEE - logit link *N* = 2 (5.0) [17, 27]	Continuous	Binary	Non-linear relationships considered through piecewise models or splines, *N* = 1 (2.5) [17]	To attempt to include a variety of shapes of relationships in the model using data from all time points	Not applicable	Includes all measured values of longitudinal variable with various relationships with risk	Splines harder to interpret; Produces population averages not individual predictions
	Continuous	Binary	Multiple time points, N = 1 (2.5) [27]	To include values and change at all time points	Not applicable	Includes all measured values of longitudinal variable	Produces population averages not individual predictions
GEE – log link, N = 2 (5.0) [22, 37]	Continuous	Rates	Multiple time points, *N* = 1 (2.5) [37] Multiple time points categorized as stable, increasing (in the second or third time point), decreasing, unstable, *N* = 1 (2.5) [22]	To include all time points in predicting rates	Not applicable	Includes all measured values of longitudinal variable	Produces population averages not individual predictions
Poisson regression, *N* = 2 (5.0) [23, 26]	Continuous	Rates	Baseline only, *N* = 2 (5.0) [23, 26]	To enable modelling of baseline rate	Not applicable	Enables modelling of baseline rate in a parametric manner	Dependence between measurement times is ignored
Linear Mixed Effects model, N = 4 (10.0) [33, 44, 46, 96]	Continuous, categorical	Continuous	Repeated measures, N = 4 (10.0) [33, 44, 46, 96]	To predict changes over time	Random effects are independent of covariates	Includes all measured values of longitudinal variable	None
Fixed effects linear regression, *N* = 1 (2.5) [52]	Continuous, categorical	Continuous	The variable is transformed by subtracting patient-level mean to remove between patient variation. *N* = 1 (2.5) [52]	To predict changes over time	Not applicable	Includes all measured values of longitudinal variable; Relaxes assumption of independence of random effects from covariates; Computationally very easy to fit compared with mixed effects models	Lower statistical efficiency than mixed effects models

PH - Proportional Hazards

^a^ Average BMI _total_ = ((BMI-67 x time_I-II_) + (BMI-85 x time_II-III_) + (BMI-96 x time_III_-))/time_total_

Total weight change = (((BMI-67 - BMI-85) x time_I-II_) + ((BMI-85 - BMI-96) x time_II-III_))/time_I-III_.

BMI deviation = absolute value of (BMI-85 - (BMI67 + BMI-96)/2).

^b^ Calculated as the average daily risk of either hypoglycemia or hyperglycemia

The simplest method of utilizing the Cox PH model was used by including the values of the longitudinal outcome at baseline (Time 0) (n = 7, 17.5%) [18, 21, 24, 43, 50, 53, 54]. For example, Tanne et al. used baseline values of SBP to predict ischemic stroke mortality [53]. This model is easily interpretable clinically; it only uses data from a single time-point per patient and does not take into account all available data. Clustering and meta-analysis techniques were also incorporated through the Cox PH model. A study using impaired sleep as a CVD risk factor included patients in two separate baseline waves. Patients could appear in both waves and clustering was accounted for when fitting the Cox PH model [24]. A study examining the association between cholesterol and cardiovascular mortality fitted Cox PH models for each year of follow-up, and combined the coefficients from these models using meta-analysis techniques [50].

Three (7.5%) studies included the difference between the longitudinal predictor at baseline and a previous value as a covariate in the Cox model, [28, 35, 38] for example, risk of coronary heart disease was predicted by using the difference between a patient’s current Framingham Risk Score and their score 3 or 6 years ago [35]. This is a simple measure; however, it assumes that change is linear between two time-points. Further, three (7.5%) studies used a slope to predict CVD and the slope was calculated manually by dividing the difference by time duration [25, 29, 32]. Other summaries were included in the Cox PH models as covariates such as a mean, [36] mean change, [36] standard deviation, [19] summaries of changes between categories [20, 34, 41] and stability in categories [20, 34, 41].

Six studies (15.0%) included longitudinal predictors as time-dependent covariates in the Cox model [39, 42, 45, 47, 49, 51, 55] by splitting the timescale at each time point when predictors are updated. Reinikainen et al. included time-dependent summary measures as time-dependent covariates; updated mean values and the change between the current and previous time-points for SBP, total cholesterol and current smoking status [39].

Three studies (7.5%) used logistic regression to model a binary disease outcome [30, 31, 48]. One included the predictor at baseline, [31] another compared the predictive power at multiple time points to predict risk of myocardial infarction by including them in separate models, [30] while the third used summary measures (mean (SD), mean change from baseline, range and average daily risk range) of blood glucose to predict mortality in myocardial infarction patients [48].

Four (10.0%) studies used generalized estimating equations (GEE) to model a disease outcome. Two had binary outcomes, [17, 27] while two others modelled rates [22, 37]. Of the four studies, two used a logit link [17, 27] and two used a log link [22, 37]. All four included data from multiple time points. One of the studies used summaries of changes in socioeconomic status and lifestyle habit variables between categories such as stable, increasing (in the second or third time point), decreasing or unstable, to predict the Framingham Risk Score [22].

Two studies included baseline values of the longitudinal predictor in a Poisson regression model, [23, 26] a form of Generalized Linear Model (GLM) that can be used as a fully parametric alternative to the Cox PH model. Poisson regression for survival analysis involves splitting the follow-up time into intervals and assuming a constant baseline hazard in each interval [97].

Four (10.0%) studies modelled changes in risk scores over time using linear mixed effects (LME) models, [33, 44, 46] for example, predicting the trajectory of the Framingham Risk Score over four time-points [44]. Fixed effects linear regression was used by one study [52] to examine how change in body mass index (BMI) is correlated with the Framingham Risk Score.

### Two-stage models

A two-stage modelling approach was used in 29 (36.3%) studies (Table 4) [57–85]. In a two-stage approach, the longitudinal data is first summarized with a longitudinal model(s). Parameters and/or estimates from this/these model(s) are then included as covariates in a survival model. The Cox PH model was used in most studies (n = 26, 89.7%) [57, 58, 60–63, 65–73, 75–78, 80–85, 88]. A weakness of the two-stage approach is that uncertainty in the longitudinal data summaries produced in the first stage is ignored.

**Table 4:** Summary of two stage approaches used to incorporate longitudinal data in survival models

Method N(%)[refs]	Longitudinal outcome type	Disease outcome type	How the longitudinal data were used in the analysis N (%) [refs]	Reason for the use of method	Assumptions	Pros	Cons
Cox model, N = 26 (89.7) [57, 58, 60–63, 65–73, 75–78, 80–85, 88]	Continuous	Time to event	Summary statistics from linear regression, *N* = 9 (31.0) [57, 62, 63, 71, 78, 80, 82, 83, 85] Slope and/or coefficient of variation, N = 8 (27.6) [62, 63, 71, 78, 80, 82, 83, 85] Slope, *N* = 1 (3.4) [57]	To incorporate a constant change or variation in the survival model	PH; Change is linear	Incorporates information from all time points	Does not allow for adjustment by other covariates as it cannot calculate overall coefficients
	Continuous, Categorical	Time to event	Latent class model used to calculate trajectory of longitudinal variable, *N* = 17 (24.4) [58, 60, 61, 65–70, 72, 73, 75–77, 81, 84, 88]	To find groups for the trajectories based on the data	PH; Population of trajectories arises from a finite mixture	Very effective at summarizing trajectories	Cannot place patients into trajectory groups easily in clinical practice; Computationally very hard model to fit
Logistic Regression, N = 1 (3.4) [64]	Continuous	Binary	Latent class model used to calculate trajectory of longitudinal variable, N = 1 (3.4) [64]	To find groups for the trajectories based on the data	Population of trajectories arises from a finite mixture	Very effective at summarizing trajectories	Cannot place patients into trajectory groups easily in clinical practice; Computationally very hard model to fit
Weighted pooled logistic regression, *N* = 1 (3.4) [59]	Continuous	Binary	Inverse probability weights calculated for each time-point. Each time-point had its own logistic regression model. Model results were pooled to produce HRs, *N* = 1 (3.4) [59]	To adjust for time-varying confounders	None considered	Accounts for variation in longitudinal data; Efficient to fit	Complex model that is not easy to understand or interpret
Poisson regression, N = 1 (3.4) [79]	Continuous	Rates	Latent class model used to calculate trajectory of longitudinal variable, *N* = 1 (3.4) [79]	To find groups for the trajectories based on the data	Population of trajectories arises from a finite mixture	Very effective at summarizing trajectories	Cannot place patients into trajectory groups easily in clinical practice; Computationally very hard model to fit

HR – Hazard Ratio; PH - Proportional Hazards

Two methods were commonly used to generate summaries from longitudinal data to include in a Cox PH model as a covariate. The simplest method calculated summary measures such as a slope or the coefficient of variation (equivalent to residual variance) using a linear regression model for each patient in nine studies (31.0%) [57, 62, 63, 71, 78, 80, 82, 83, 85] Gao et al. used linear regression to estimate the intercept, slope, square of the slope and coefficient of variation for blood pressure that were then included in a Cox PH model to assess how variation and changes in blood pressure were associated with mortality [63].

The second most frequently used method (n = 17, 58.6%) was group-based trajectory models (GBTMs) to model the trajectory of the longitudinal variable [58, 60, 61, 65–70, 72, 73, 75–77, 81, 84, 88]. Wang et al. identified four separate trajectories of sleep duration and used these to predict risk of cardiovascular events or mortality [69]. Most models were fitted using the Proc Traj package from SAS [98] (*n* = 10, 58.8%), [60, 65–70, 73, 75, 81] although other software, including Stata (traj) [99] and R (lcmm) can be used [100]. Trajectory groups from GBTMs were also used in logistic regression (n = 1) [64] and Poisson regression (n = 1) [79] analyses of survival outcomes.

Desai et al. used weighted pooled logistic regression with inverse probability weights (IPWs) to examine the association between changes in serum uric acid and risk of incident diabetes, CVD and renal decline [59]. These models are complex, but the resulting hazard ratios can be interpreted as causal estimates assuming no unmeasured confounders [101].

### Joint models

A joint modelling approach, where both the longitudinal variable and the survival model are fitted simultaneously, was used for eight studies (10.0%) [86, 87, 89, 91–93]; (Table 5). This approach makes full use of the available data and may be more statistically efficient than fitting a two-stage model; however, this increases the computational complexity.

**Table 5:** Summary of joint modelling approaches used to incorporate longitudinal data and survival data

Method N(%)[refs]	Longitudinal outcome type	Disease outcome type	How the longitudinal data were used in the analysis N (%) [refs]	Reason for the use of method	Assumptions	Pros	Cons
Frequentist joint model, N = 6 (75.0) [86, 87, 89, 91–93]	Continuous	Time to event	Longitudinal data were modelled in LME. Survival data were modelled in Cox PH. N = 5 (62.5) [86, 87, 91–93] Association structures: Current value, *N* = 2 (25.0) [86, 93] Current value and 1st derivative, N = 2 (25.0) [91, 92] 1st derivative, *N* = 1 (12.5) [87]	To predict changes in risk score over time using repeated measures	None considered	Includes all measured values of longitudinal variable	Computationally very hard model to fit
	Continuous	Time to event	Structured equation model incorporated in survival model as covariate, *N* = 1 (12.5) [89]	To incorporate a constant change or variation in the survival model	PH; Change is linear	Incorporates information from all time points	Does not allow for adjustment by other covariates as it cannot calculate overall coefficients
Latent class model, N = 1 (12.5) [88]	Continuous	Time to event	Latent class model used to calculate trajectory of longitudinal variable. Trajectory class incorporated in model as covariate, *N* = 1 (12.5) [88]	To find groups for the trajectories based on the data	PH; Population of trajectories arises from a finite mixture	Very effective at summarizing trajectories	Cannot place patients into trajectory groups easily in clinical practice; Computationally very hard model to fit
Bayesian approach, N = 1 (12.5) [90]	Ordinal	Time to event	Item response theory models were used to model ordinal data from a multi-question survey using a latent parameter. This latent parameter was modelled using a linear growth model and was incorporated in a multi-state Gompertz survival model as a covariate, *N* = 1 (12.5) [90]	To model ordinal survey data with the correct distribution	Values constant between observations	Incorporates data from complex survey accounting for ordinal data modelling the data directly rather than modelling the sum of the responses	Complex and requires Bayesian code to be used to define the model

LME - Linear Mixed Effects; PH - Proportional Hazard

.

Five studies (62.5%) [86, 87, 91–93] modelled the longitudinal outcome using an LME model and the survival outcome using a Cox PH model. One study used the model to analyze the association between blood pressure and coronary artery disease [92].

Batterham et al. used latent growth models, which is similar to LME models, to predict the slope and intercept of five different cognitive tests jointly with a Cox PH model to predict the risk of all-cause mortality and cause-specific mortality. The model is fitted using Mplus [89]. Ogata et al. used a GBTM jointly with a Cox PH model to predict risk of CVD using trajectories of fasting plasma glucose [88]. van den Hout et al. used a Bayesian approach to jointly model ordinal data from the Mini-Mental State Examination. Item response theory (IRT) models were used to model the ordinal data before using Gompertz survival models to model a multi-state outcome (e.g. healthy, history of strokes and death) [90].

## Discussion

This review has identified a multitude of methods to analyze the risk of CVD using longitudinally repeated data. There has been an increase in the complexity of methodology used over the past two decades, with an increasing proportion of studies applying more efficient approaches such as two-stage and joint models over time. However, many studies only used simple analysis based on one time-point, even when more data were available.

When CVD risk was modelled in a two-stage model, two methods were commonly used: patient-level linear regression to account for longitudinal data, followed by the Cox PH model to estimate CVD risk, or GBTMs followed by the Cox PH model. On the other hand, in a joint model, the longitudinal and survival data are modelled simultaneously. Both models aimed to utilize a patient’s time-varying risk factors to predict CVD risk. These models can provide an important understanding of the association between changes in risk factors over time and CVD risk, which can be used to influence risk management decisions.

The characteristics and assumptions of a model need to be considered carefully when selecting and interpreting models. Although a time-dependent covariate Cox PH model provides an advantage by enabling risk estimates to be updated during follow-up for new individuals, the model assumes that values are constant between two time-points and are measured without error. Computationally, the model can quickly become unfeasible to fit if predictor values are updated at different time points for each individual. This model is also prone to greater overfitting as a time-dependent covariate forms a complex function over time which could lead to too much modelling; hence, this should be used with caution [102].

The disease risk is estimated as an odds ratio from logistic regression, and it should be interpreted appropriately (not as a risk ratio), especially when the outcome is not rare. Odds ratios cannot be compared between datasets or models with different independent variables because they reflect unobserved heterogeneity between observations which varies between datasets and models [103].

Three different methods to model within-patient variation with a continuous outcome were encountered: GEEs, LME models and fixed effects regression. GEEs are an extension of GLMs that allows a correlation structure between observations [104, 105]. Similarly to GLMs, using different link functions or distributions, GEEs can be used to model continuous, binary, count or binomial outcomes. LME models are an alternative for continuous outcomes, which assumes that the residual error is normally distributed and models within-patient correlation with random effects which are also assumed to be normally distributed and independent of covariates. This allows LME models to make individual patient predictions rather than just the population-level predictions from a GEE [106]. Fixed effects regression relaxes the assumption that random effects are independent of covariates. The model is computationally easier to fit than an LME model and is more appropriate if unobserved heterogeneity is correlated with covariates [107].

GBTMs are a form of a finite mixture model that is an effective way of identifying a fixed number of groups of individuals who follow similar trajectories [108]. However, they are computationally difficult to fit. The results of this model may also be difficult to apply in clinical practice as it can be difficult to assign a patient to one trajectory group by hand accurately.

In a standard joint model, the longitudinal outcome is modelled by an LME model and the survival outcome by a Cox PH model. The two outcomes are linked via shared random effects to capture the time-dependent association between longitudinal measurements and the risk of an event [109]. This association can be defined in a variety of ways, but common approaches include a linear predictor (i.e. current value), a derivative (i.e. rate of change) or an integral (i.e. cumulative effect) of the linear predictor.

The reasons for the slow increase in the utilization of two-stage and joint models is multi-factorial. Computationally these models can be much harder to fit than single-stage models, with joint models in particular conveying significant computational burden. Also, there is poor awareness of inefficiency in simple methods. Many studies may not include a statistician as part of the research team and therefore, authors may not have the requisite experience of analyzing longitudinal data. However, as these methods become more common, and software to fit the models becomes more accessible and computationally more powerful, the utilization of more efficient methods should increase over time.

Different risk prediction models are appropriate for different settings. Models may be used for prediction in a clinical setting or used for studying the association between an exposure and an outcome. Many risk prediction models require computation to obtain a precise risk prediction which poses difficulties in a clinical setting. Existing risk prediction models such as QRISK3 use online calculators to predict risk using a complex model. Inputting all longitudinal data into an online calculator may not be possible in a clinical setting. Alternatives include either using single-stage models including summaries of the longitudinal data such as means, slopes or differences or integrating the risk prediction model into EHRs software. More complex models such as two-stage or joint models are very useful for explaining associations although interpretation can require more thought. Joint models especially need greater consideration when interpreting association structures such as random effect associations. Assigning and interpreting complex groups for GBTMs can be difficult for clinicians in practice although it is sometimes possible to assign clear descriptions to GBTM groups such as high, low, increasing or decreasing.

Reporting of the data in the included studies was highly variable. For example, the number of time-points used per patient in each study was disparate with studies choosing from a selection of mean, median, a range (e.g. 3–5), the maximum possible or frequency over the follow-up period; some studies, especially studies based on electronic health records, did not report the number of time-points, resulting in difficulties ascertaining exactly how many measurements were used. Follow-up length was also described as a date range, mean, median, maximum, dates of study waves etc. This resulted in a loss of clarity, especially when studies had a separate follow-up period for longitudinal data collection and for the survival outcome. Also, some studies did not report variables removed as part of variable selection.

### Strengths and limitations

This review examined all available studies that have assessed the relationship between the trajectory of longitudinal risk factors and the risk of a cardiovascular event or mortality, and summarized the methods used in analyzing longitudinal risk factors for CVD risk. This review can be readily used to identify methods for future analysis of longitudinal trajectories and risk prediction in CVD. However, due to search terms having this specific focus, single-stage models underutilizing the data available are more likely to be underrepresented.

Queries over eligibility or the article content were thoroughly discussed among the authors of this review before reaching the final decision. However, articles were searched and screened by one author and there remains a possibility of bias or error. This review focused solely on a search of MEDLINE-Ovid providing a focused and consistent search, although inclusion of other bibliographic databases may have returned other studies.

This review was designed to highlight the strengths of statistical methods for summarizing longitudinal data to predict CVD risk. A deeper comparison of the methods using simulated data have been discussed in the literature numerous times as the methods were first developed or in their application [110–112]. A machine learning approach may also be worth considering when designing a study, although our search only identified one study using machine learning methods [113]. Machine learning algorithms have the potential to provide stronger predictions of risk using many variables; however, this incurs greater potential for overfitting and collinearity between variables. To avoid this, machine learning applies a greater focus on increased model validation, preferably external validation [114].

## Conclusions

The use of two-stage and joint models is a critical part of understanding the relationship between the longitudinal risk factors and CVD. Many studies still employ single stage approaches which often underutilize available longitudinal data when modelling cardiovascular risk. Further studies should aim to optimize the use of longitudinal data by using two-stage and joint models whenever possible for a more accurate estimation of cardiovascular risk.

## Supplementary Information

**Additional file 1.**

## Data Availability

The dataset(s) supporting the conclusions of this article is(are) included within the article (and its additional file(s)).

## Abbreviations

BMI: body mass index
CVD: cardiovascular disease
GBTM: group based trajectory model
GEE: generalized estimating equations
GLM: generalized linear model
IPW: inverse probability weight
IRT: item response theory
LME: linear mixed effect
PH: proportional hazards
SBP: systolic blood pressure
