# Supplementary material for: Modelling of longitudinal data to predict cardiovascular disease risk: a methodological review
Source: BMC Med Res Methodol. 2021 Dec 18;21:283. doi: 10.1186/s12874-021-01472-x (PMC8684210; doi:10.1186/s12874-021-01472-x)
Supplement: Supplementary file 1 — Additional file 1. [file 12874_2021_1472_MOESM1_ESM.docx]

**Supplementary Table 1.** MEDLINE-Ovid Search strategy

| Search No. | Search term |
| --- | --- |
| 1 | longitudinal.af. |
| 2 | repeat*measure*.af. |
| 3 | hierarchical.af |
| 4 | multilevel model*.af |
| 5 | change.af. |
| 6 | slope.af. |
| 7 | profile.af. |
| 8 | trajector*.af. |
| 9 | growth curve.af. |
| 10 | cardiovasc*.ti. |
| 11 | cerebrovasc*.ti. |
| 12 | atrial fibrillation.ti. |
| 13 | coronary.ti. |
| 14 | artery.ti. |
| 15 | disease.ti. |
| 16 | 14 or 15 |
| 17 | 13 and 16 |
| 18 | stroke.ti. |
| 19 | exp *Myocardial Infarction/ |
| 20 | *Cardiovascular Diseases/ |
| 21 | exp *Coronary Disease/ |
| 22 | exp *Stroke/ |
| 23 | *Brain Ischemia/ |
| 24 | *Heart Diseases/ |
| 25 | exp *Intracranial Hemorrhages/ |
| 26 | exp *Intracranial Hemorrhage, Traumatic/ |
| 27 | 25 not 26 |
| 28 | 10 or 11 or 12 or 17 or 18 or 19 or 20 or 21 or 22 or 23 or 24 or 27 |
| 29 | 1 or 2 or 3 or 4 |
| 30 | 5 or 6 or 7 or 8 or 9 |
| 31 | 28 and 29 and 30 |
